# Electric Field Control Of Moiré Skyrmion Phases in Twisted Multiferroic NiI_2_ Bilayers

**DOI:** 10.1021/acs.nanolett.4c04582

**Published:** 2024-11-22

**Authors:** Tiago V. C. Antão, Jose L. Lado, Adolfo O. Fumega

**Affiliations:** † Department of Applied Physics, Aalto University, 02150 Espoo, Finland

**Keywords:** 2d multiferroics, skyrmions, van der Waals heterostructures, Twisted bilayer, moiré, frustrated magnetism

## Abstract

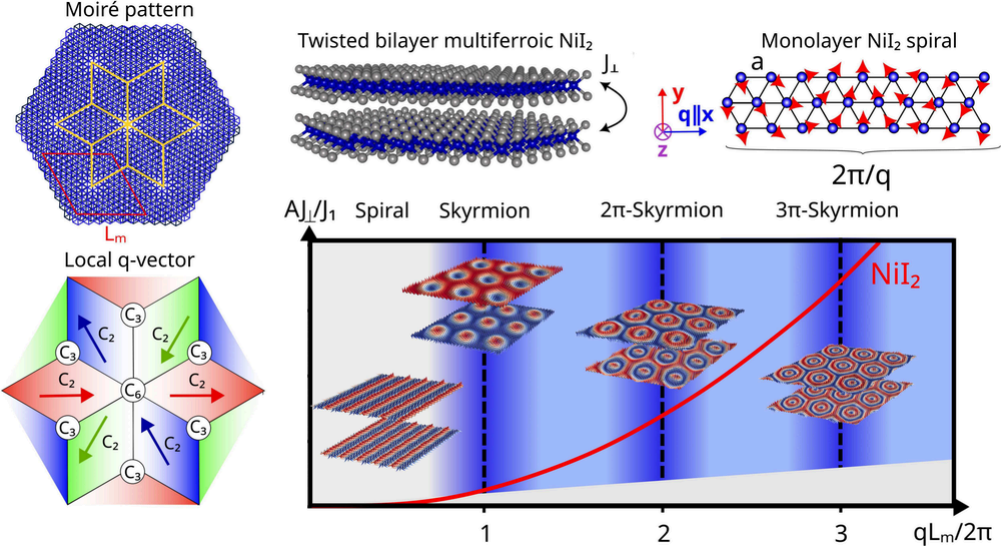

Twisted magnetic van der Waals materials provide a flexible platform to engineer unconventional magnetism. Here we demonstrate the emergence of electrically tunable topological moiré magnetism in twisted bilayers of the spin-spiral multiferroic NiI_2_. We establish a rich phase diagram featuring uniform spiral phases, a variety of *kπ*-skyrmion lattices, and nematic spin textures ordered at the moiré scale. The emergence of these phases is driven by the local stacking and the resulting moiré modulated frustration. Notably, when the spin-spiral wavelength is commensurate with the moiré length scale by an integer *k*, multiwalled skyrmions become pinned to the moiré pattern. We show that the strong magnetoelectric coupling displayed by the moiré multiferroic allows electric control of the *kπ*-skyrmion lattices by an out-of-plane electric field. Our results establish a highly tunable platform for skyrmionics based on twisted van der Waals multiferroics, potentially enabling a new generation of ultrathin topologically protected spintronic devices.

Skyrmions are topologically protected magnetic structures that have garnered significant attention due to their potential applications in spintronics, particularly in the areas of data storage and manipulation.^1−4^ These nanoscale spin textures are of great interest for advanced energy-efficient memory technologies because of their stability and unique spin configurations. Skyrmions have been observed or proposed in a variety of systems, including two-dimensional (2D) magnets,^5−8^ magnetic thin films,^9^ and material interfaces.^10^ Their formation is typically driven by mechanisms such as the Dzyaloshinskii-Moriya (DM) interaction,^11,12^ magnetic frustration,^13−17^ and other types of magnetic anisotropy modulation.^18^ Despite the excitement surrounding skyrmions, their emergence in insulating materials is relatively rare.^19−21^ This presents a challenge for integrating skyrmions into insulating platforms, which are particularly attractive for low-power spintronic applications.

Two-dimensional van der Waals (vdW) materials provide an exciting new avenue for engineering exotic quantum phases of matter,^22^ including the design of exotic magnetic phases.^23−25^ The weak vdW bonding between the layers in this class of materials allows easily reach their monolayer limit, establishing a rich family of 2D building blocks.^26−34^ The monolayers can be stacked together to generate heterostructures with emergent collective states.^35−37^ Moreover, a twist angle can be introduced between stacked layers, giving rise to a moiré length scale that drives the emergence of complex phase diagrams.^38,39^ For example, twisting has been shown to induce multiferroicity and skyrmionic patterns in bilayer chromium trihalides (CrX_3_, X = Br, I, Cl)^40−47^ and transition metal dichalcogenides.^48,49^ Additionally, exotic magnetic orders, including whirls and stripes, have been proposed in twisted 2D magnets like RuCl_3_.^50^ The isolation of monolayer NiI_2_,^51,52^ has recently provided a new avenue for engineering matter by introducing multiferroic behavior as a new building block within magnetic vdW materials.^53−55^ Multiferroic materials exhibit simultaneous electric and magnetic order.^56,57^ In particular, NiI_2_ is a type-II multiferroic whose ferroelectricity originates from its helical magnetic order and strong spin–orbit coupling^58^ resulting in a strong magnetoelectric coupling.^52,59^ It has been reported that the multiferroic order in NiI_2_ can be tuned by external factors such as strain,^60^ pressure,^61,62^ substrate engineering^63^ or cobalt substitution.^64^ However, the emergence of exotic magnetic orders displaying strong magnetoelectric coupling in NiI_2_ moiré heterostructures has remained unexplored.

In this paper, we demonstrate that twisted bilayer NiI_2_ allows engineering topological magnetic textures, realizing a whole new family of multiferroic orders. We show that the moiré created by the twist angle enhances the frustration in the system and produces a variety of exotic magnetic orders, specifically exotic *kπ*-skyrmions driven by the commensurability of the spin spiral wavelength and moiré length scale. Additionally, we show that the multiferroic order of twisted NiI_2_ provides an unprecedented level of control over skyrmion lattice states using external electric fields, allowing driving the system between different skyrmionic configurations. We provide a phase diagram for the skyrmion phases in the twisted bilayer and demonstrate the electric-field tunability of these magnetic states, establishing twisted bilayer NiI_2_ as a versatile platform for engineering and manipulating topological spin textures.

In monolayer NiI_2_, multiferroicity emerges from the coexistence of a noncollinear magnetic spin-spiral order and strong spin–orbit coupling^58,65^ via the inverse Dzyaloshinskii-Moriya (DM) effect.^66−68^ This effect causes a noncentrosymmetric displacement of electric charge due to the noncollinear spin structure leading to an electric polarization. The magnetic ground state of monolayer NiI_2_ corresponds to a coplanar spin spiral magnetic order (Figure 1(a)). The spin spiral phase arises from the magnetic frustration induced by the competition between ferromagnetic first-neighbor *J*_1_ and antiferromagnetic third-neighbors *J*_3_ magnetic exchange interactions^52,69,70^ (Figure 1(b)). In the bulk limit, additional interactions, such as the Kitaev anisotropic interaction and biquadratic exchange, have also been proposed to influence the ground state spin configuration in NiI_2_.^71^ However, in the ultrathin limit, theoretical analyses have found these terms to be negligible,^63^ and atomic-scale experiments have shown that a minimal classical Heisenberg *J*_1_ – *J*_3_ model suffices to accurately describe the observed ground state.^52^ The magnetization in the spin-spiral phase of monolayer NiI_2_ is described by propagation vector ***q***, determining the direction and wavelength 2π/*q* of the spin-spiral. The ***q*** vector is intrinsically constrained by the exchange interactions of the spin Hamiltonian to the direction of the first (or third) neighbors and its periodicity is determined by the *J*_3_/*J*_1_ ratio.^72,73^ In particular, *J*_3_ ≈ – 0.3*J*_1_ is found to reproduce the experimentally observed spiral periodicity.^52^ In addition, experimentally, the magnetization is observed to be mostly in-plane,^52^ which is accounted for by an in-plane anisotropy term *A*_*z*_ = −0.02*J*_1_, which fixes the rotation plane as , as depicted in Figure 1(a). Other additional terms such as anisotropic symmetric exchange interactions may also play a role in fixing the direction of ***e*^** as well as changing the length-scale determined by the *J*_3_/*J*_1_ ratio,^20^ however these should not contribute significantly to the results presented in the following discussion (see the S.I. for a detailed discussion). When two NiI_2_ layers are stacked, an antiferromagnetic interlayer exchange *J*_⊥,(*ij*)_ is established (Figure 1(c)). *J*_⊥,(*ij*)_ is parametrized by an exponentially decaying function peaking at the next nearest interlayer neighbor distance at zero twist angle with strength *J*_⊥_. The total Hamiltonian for twisted NiI_2_ bilayer reads
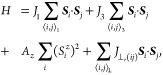
1where ⟨·,·⟩_*n*_ denotes the *n*th nearest neighbors and ⟨·,·⟩_⊥_ refers to summation over sites in distinct layers. The emergence of distinct orders in the twisted bilayer arises from the additional frustration introduced by interlayer coupling (Figure 1(c)). Note that, as opposed to other twisted magnets,^40,41,50^ the sign of *J*_⊥_ does not have a stacking dependence, and the emerging orders in this system will be driven by the interplay between the spin-spiral and moiré length scales. A detailed *ab initio* analysis of the stacking dependence of *J*_⊥_ in twisted NiI_2_ is provided in the S.I. Physically, the presence of antiferromagnetic interlayer interactions causes a local antialignment of spins on the top and bottom layers resulting in the two layers having the same direction and magnitude of ***q***. The first effect of Figure 1(d) transitions from a ferromagnetic phase to a spin-spiral phase at a ratio of *J*_3_/*J*_1_ = 0.25 when *J*_⊥_ = 0, and this critical value is enhanced for *J*_⊥_ > 0. The value of the ratio *J*_3_/*J*_1_ for twisted bilayer NiI_2_ is taken to be approximately the same as the monolayer, and the interlayer exchange is estimated to be given by *J*_⊥_/*J*_1_ ≈ – 0.14,^59,71^ marked in the phase diagram Figure 1(d).

**Figure 1:**
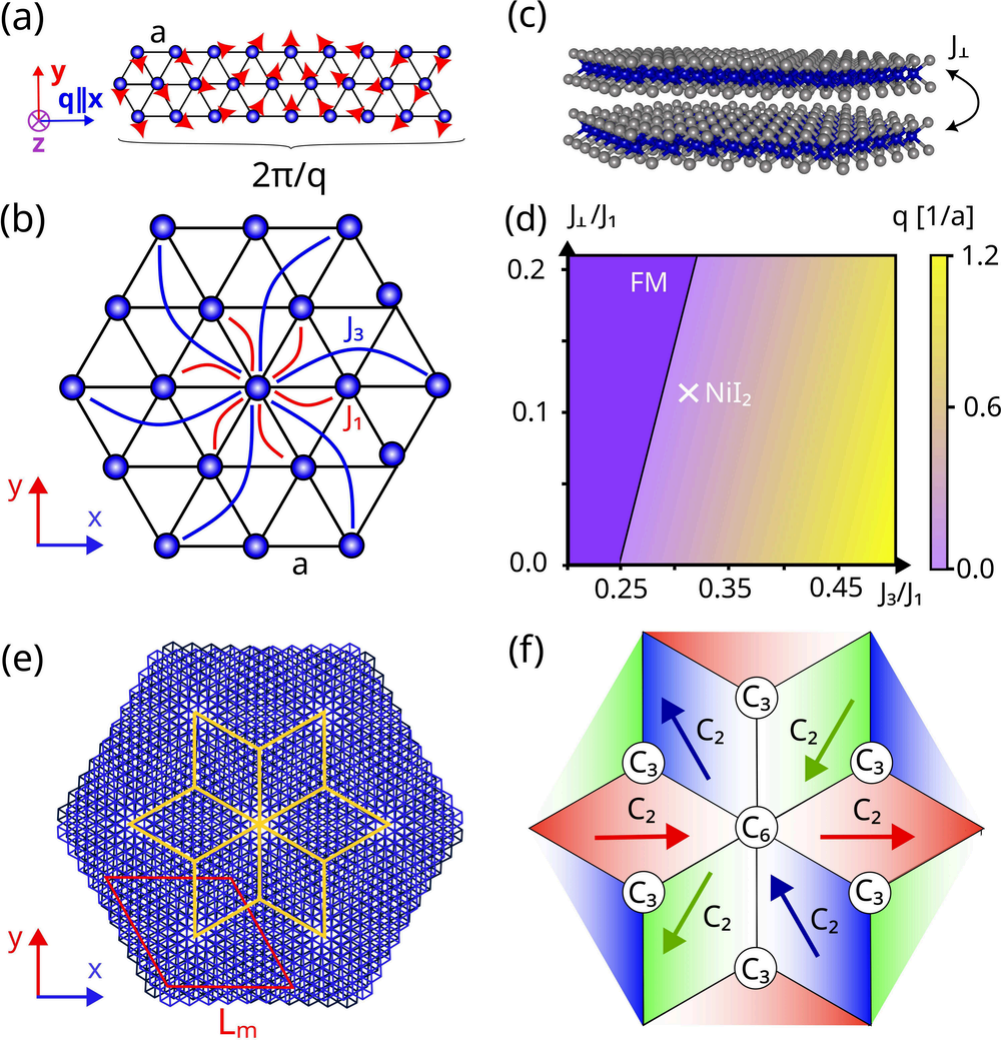
(a) Schematic of the spin-spiral of monolayer NiI_2_ with wavelength 2π/*q*.(b) Exchange interactions *J*_1_ and *J*_3_ of the Ni triangular lattice leading to the noncollinear spin-spiral state. (c) Schematic of a NiI_2_ twisted bilayer, with Ni atoms represented in blue, and I atoms in gray. (d) Magnitude of the propagation vector *q* as a function of the interlayer exchanges *J*_⊥_/*J*_1_ and *J*_3_/*J*_1_. (e) Moiré pattern of twisted bilayer NiI_2_, showing the moiré unit cell (red), and the spatial patches with *C*_2_ symmetric rhombohedral stacking along different directions (yellow). (f) Schematic of the domains of *q* originated by the local broken symmetry.

When the two layers are twisted, the phase diagram of Figure 1(d) is found to be unchanged up to twist angles of 6°, so the renormalization of the magnitude of ***q*** does not depend on the angle for small twist angles. However, the moiré pattern induces regions of higher spin density along certain spatial directions within each moiré unit cell, as illustrated in Figure 1(e). In Figure 1(f), the different colored regions correspond to areas of the moiré pattern where the rotational symmetry of the crystalline environment is broken from the monolayer *C*_6_ down to a *C*_2_ rhombohedral stacking, promoting the emergence of patches with well-defined *q*-vector directions. In the center of the moiré supercell, a *C*_6_ symmetric monoclinic stacking area remains, where six *q*-vector patches coalesce. Additional *C*_3_ symmetric regions appear, surrounded by three *q*-vector patches. This local modulation of ***q*** into a function of position ***q***_*i*_ is, contingent on the moiré supercell being large enough for these symmetries to approximately hold, as well as *J*_⊥_ being strong enough relative to the moiré unit cell size *L*_m_ to generate a sufficiently strong pinning potential. Therefore, a finite twist angle between layers will introduce an extra moiré length scale *L*_m_ that will interplay with the spin spiral length scale ***q*** of each layer, thus giving rise to exotic magnetic scales.

We move on to computing the magnetic ground states arising from the model for twisted NiI_2_ as a function of the competing spin spiral and moiré length scales. We find the lowest energy configurations under generalized twisted boundary conditions, which allows us to account for ground states where the spiral wavelength and moiré length scale are not commensurate. Two paradigmatic examples of the kind of topological magnetic phases achievable in twisted bilayer NiI_2_ are the skyrmion and skyrmionium (2π-skyrmion) lattices^6,74−76^ (see the S.I. for details on the calculation of the spin ground states). These are plotted in Figure 2(a) considering the commensurate cases *qL*_m_/2π = 1 and *qL*_m_/2π = 2, with twist angles θ ≈ 3.89° and θ ≈ 2.13° respectively. Only the top layer is showcased, although both layers are used in the calculation. The bottom layer is antiferromagnetically aligned with the top. Associated with these magnetic textures, in Figure 2(b) we computed the local ***q***. The results match well with the schematic presented in Figure 1(f), confirming that the moiré length scale induces the pinning of the ***q*** vector directions. Finally, the presence of strong spin–orbit coupling λ induces an emergent electric polarization ***P***_*ij*_=λ***r***_*ij*_ × (***S***_*i*_ × ***S***_*j*_) in noncollinear magnets.^66,67^Figure 2(c) shows the emergent polarization associated with the skyrmion and skyrmionium lattices in the top layer of the twisted NiI_2_ system. The bottom layer has an identical polarization profile. It can be seen that the emergent polarization points mostly in the out-of-plane direction ***z***. This starkly contrasts with the monolayer case and is a key feature that allows the twisted bilayer to couple to out-of-plane electric fields. In the twisted bilayer system, the microscopic mechanism that originates ferroelectric polarization is the same as in the monolayer, namely the inverse DM mechanism.^67^ However, it is the emergent noncoplanar magnetic order driven by the moiré scale frustration that results in a dominantly out-of-plane component. This is the key difference distinguishing the magnetic and ferroelectric orders in the twisted bilayer from those found in the monolayer. The novel multiferroic behavior found in the bilayer also allows for the magnetic control of ferroelectricity. This feature allows us to distinguish it from the sliding ferroelectricity emerging in other twisted systems (such as twisted boron nitride bilayers),^77^ for which the driving mechanism is the lattice distortions stemming from relaxation effects. For twisted bilayer NiI_2_, we expect that atomic relaxations will not produce large effects on the magnetic order and the expected phase diagram for the system (see the Supporting Information for details on the effect of atomic lattice relaxations on the magnetic order).

**Figure 2:**
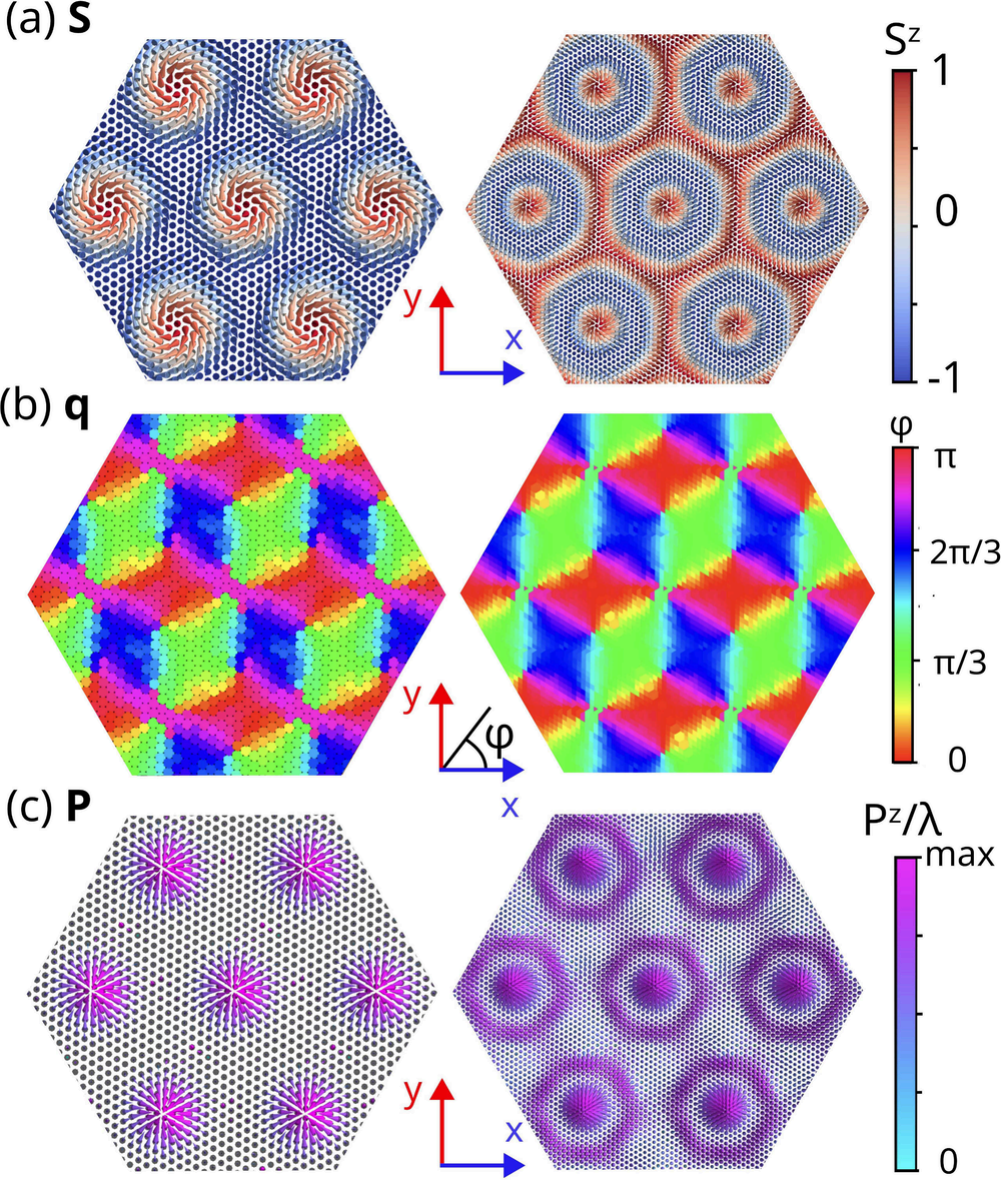
(a) Spin ***S***, (b) propagation vector ***q***, and (c) polarization ***P*** of a skyrmion and skyrmionium lattice in twisted bilayer NiI_2_. These occur for twist angles θ ≈ 3.89° and θ ≈ 2.13°, left and right panels, respectively. The panels show the top layer of a twisted bilayer, with the bottom one featuring a magnetic configuration that is antialigned with the top, and an equivalent polarization profile.

The ground-state solutions as a function of the twist angle are presented in the phase diagram shown in Figure 3(a). Variations in the twist angle and *J*_⊥_ allow for the identification of many other topologically distinct phases. This phase diagram shows many topologically distinct phases as a function of the commensurability parameter *qL*_*m*_ and the interlayer coupling *J*_⊥_. The transition into the spiral phase is found to be approximately linear when *J*_⊥_ is normalized by the moiré area ∼*L*_m_^2^, which leads to a choice of vertical axis for the phase diagram of the form *J*_⊥_*L*_m_^2^/*J*_1_*a*^2^. The spin spiral phase occurs when *J*_⊥_/*J*_1_ is small or the twist angle is large, making *L*_*m*_ too small to impact the interlayer exchange field significantly (Figure 3(b)). However, the direction of ***q*** is changed from the third neighbors to the moiré superlattice vectors. A series of other phases corresponding to *kπ*-skyrmion or so-called target skyrmion lattices emerge at integer values of *qL*_m_/2π = *k*, when the moiré unit cell accommodates an integer number of spin-spiral wavelengths (Figures 3(c,e,g)). Another family of phases corresponds to nematic skyrmion lattices (Figures 3(d,f)), which occur for twist angles that lead to incommensurate values of *q* and *L*_m_. This produces whirling textures within each unit cell and breaks translational symmetry differently for each moiré lattice vector compared to the commensurate case. Therefore, these nematic phases can host coexisting skyrmion and spiral features.

**Figure 3:**
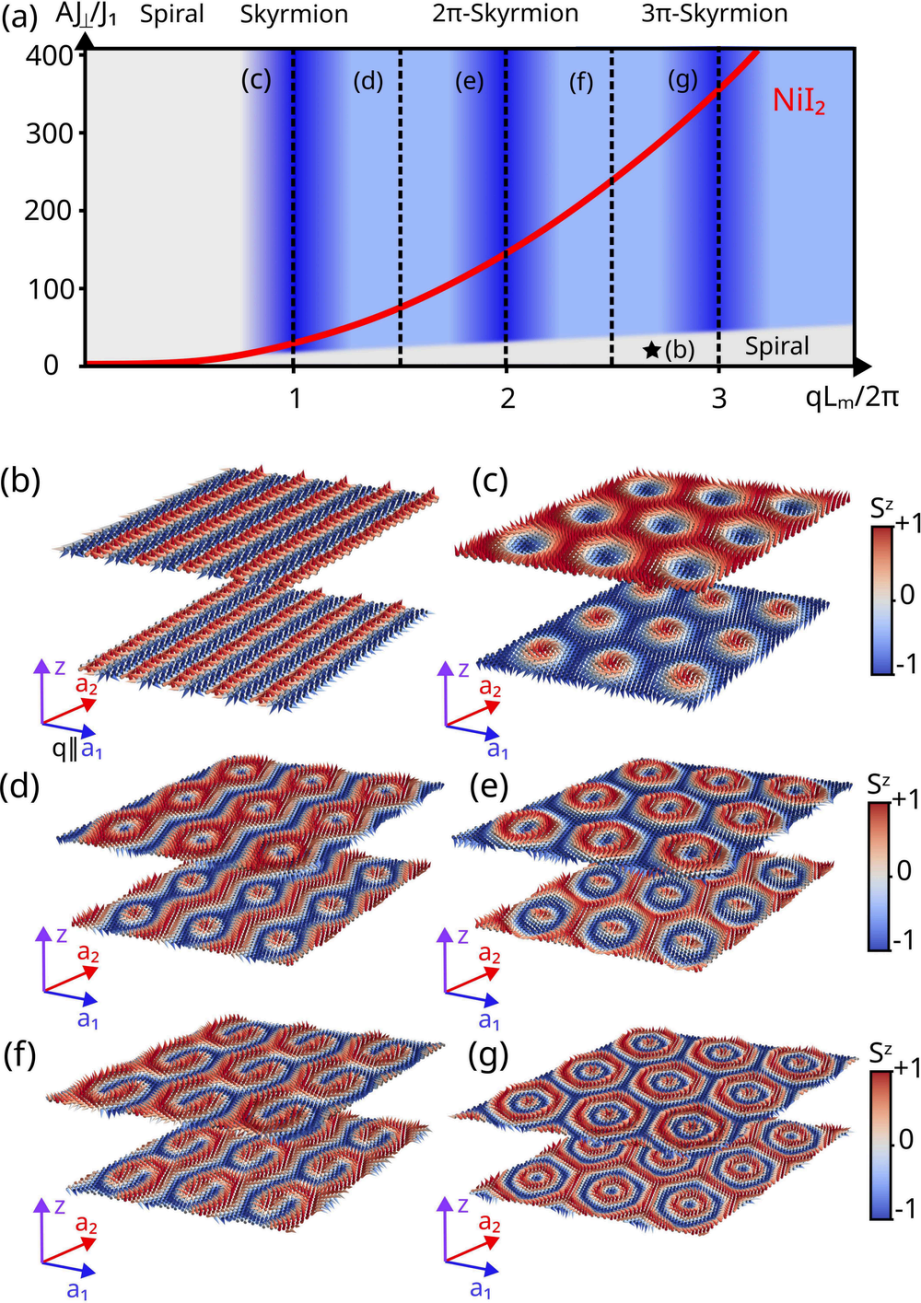
(a) Phase diagram of twisted bilayer NiI_2_ as a function of normalized interlayer exchange *AJ*_⊥_/*J*_1_ and *qL*_m_/2π. The factor *A* = *L*_m_^2^/*a*^2^ introduces the relative area between the moiré and the single unit cells, ensuring that the same ground states are represented along vertical lines in the phase diagram. The red line represents NiI_2_ as *L*_m_ increases or, equivalently, as the twist angle becomes smaller. (b-g) Representative ground state solutions of the moiré spin-spiral multiferroic bilayer phase diagram shown in panel (a): (b) uniform spin spiral; (c), (e), and (g) the simple, 2π- and 3π-skyrmion lattices, respectively; and (d) and (f) intermediate phases with nematic order between commensurate solutions.

It is worth noting that the distinct emergent phases can be distinguished based on topological features. In particular, the topology of the configuration depends on the distribution of the ferromagnetic chains and how they accommodate the local *q*-vector. These chains can form concentric loops corresponding to skyrmionic textures, or cross the borders of the moiré unit cell, leading to helical and nematic textures. For noninteger *qL*_m_ values, for instance, chains are deformed in such a way as to form horseshoe patterns, or other distinct whirls, locally breaking the described *q* patches into smaller regions, but preserving some of the global topological features of the spin configuration, such as the total winding *kπ*. In general, the textures found in between *kπ* and (*k* + 1)π Skyrmion lattices can have any winding  with 0 ≤ *p* < *k* + 1. The presence of these topological features points to potential stability under external perturbations.

*Electric field control:* We now address the effect of an external out-of-plane electric field on the ground-state spin configuration in these *kπ*-skyrmion lattice phases. The strong magnetoelectric coupling in multiferroics is particularly attractive to control skyrmion lattices. An external uniform electric field ***E***_ext_ couples to the associated electric polarization ***P***_*ij*_ generated via the inverse DM. This introduces a magnetoelectric coupling term in the Hamiltonian of the form *H*_ME_ = −∑_⟨*ij*⟩_***E***_ext_·***P***_*ij*_. The microscopic formula of the electric polarization leads to a term of the form
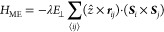
2

For , this DM term promotes the canting of spins, favoring skyrmion formation, thus enhancing the formation of Néel-type skyrmion lattices over Bloch-type counterparts. Moreover, for a specific moiré length scale *L*_m_, smaller values of *q* minimize the contribution from the polarization term, suggesting that the applied electric field can potentially alter the *kπ* value of the skyrmion lattice ground state.

For the sake of concreteness, we focus on the skyrmion lattice ground state, although all conclusions drawn here apply to higher order *kπ*-skyrmion lattices. We focus on two distinct scenarios: subjecting the bilayer system to an external electric field in an adiabatic manner and a nonadiabatic manner. Adiabatically changing the external field strength involves incremental adjustment of the field in small steps while allowing for the spin configuration to relax adiabatically. In contrast, a nonadiabatic change involves applying a sudden change to the electric field, followed by a full relaxation of the spin configuration. The results of these calculations are shown in Figure 4. An adiabatic increase of the electric field preserves the system’s topological features, such as the nature of the skyrmion lattice, while merely deforming and shifting the location of the skyrmion center, originating a horseshoe skyrmion texture. Nevertheless, the hysteretic behavior of the system reflects the emergence of these topologically equivalent yet distinctly shaped spin ground state configurations. In this sense, varying *L*_m_ may allow for a more plastic deformation of the skyrmions, whereas smaller moiré unit cell sizes restrict the allowed skyrmionic solutions leading to sharper changes in behavior (see the Supporting Information for discussion on the interplay between the length-scales set by the interlayer interaction, field-induced DM interaction, and moiré length scale).

**Figure 4:**
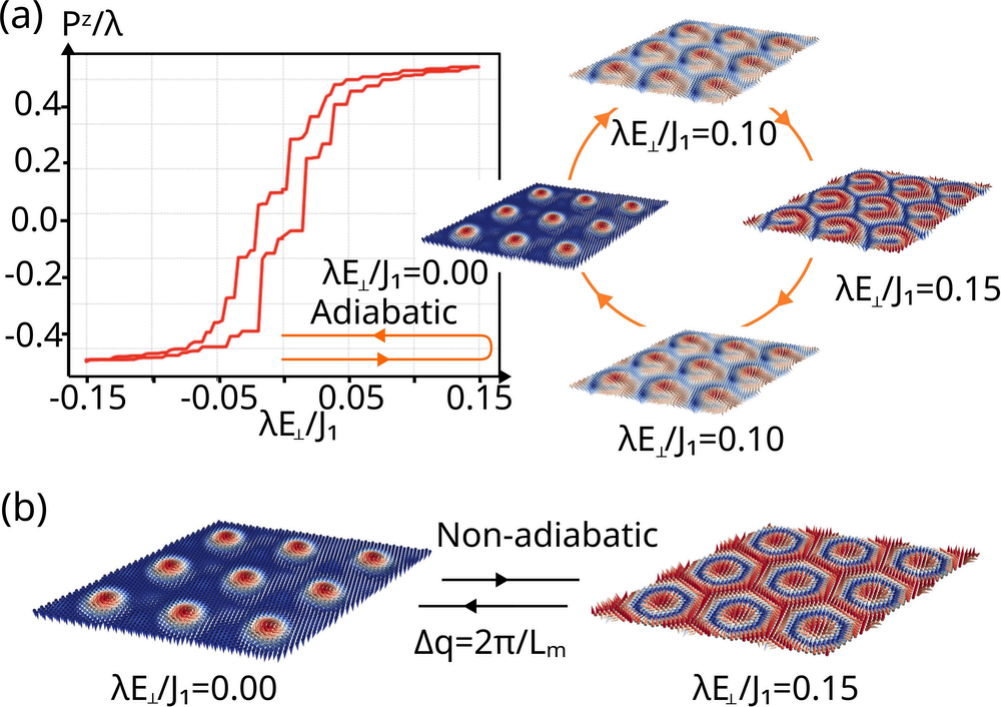
(a) Adiabatic evolution of the skyrmion lattice ground state of twisted NiI_2_. The polarization along *z* for the top layer undergoes a hysteresis loop as the field magnitude *λE*_ext_/*J*_1_ is changed along the loop 0 → 0.15 → – 0.15 → 0 (b) Nonadiabatic evolution of the ground state, resulting in a transition from a skyrmion to a skyrmionium lattice. Only the top layer is shown for clarity, with the bottom one remaining antialigned with the top.

On the other hand, nonadiabatic changes lead to more dramatic effects, altering the *k* value in a *kπ*-skyrmion. The skyrmion lattice can be transformed into a skyrmionium lattice through a nonadiabatic increase in the electric field. In this scenario, the external electric field effectively modifies the *q* of the underlying spin-spiral by Δ*q* = 2π/*L*_*m*_. Therefore, these results highlight the robustness and magnetoelectric tunability of the topological phases in twisted spin-spiral multiferroics.

We have demonstrated that twisted bilayer NiI_2_ provides a highly tunable platform for engineering a whole family of topological magnetic phases. Our results establish that twist engineering allows stabilizing a variety of magnetic orders, including skyrmion lattices and nematic phases, thanks to the interplay between interlayer coupling and the moiré pattern. The moiré-induced modulations stabilize nonuniform ***q***-vector configurations, enabling complex magnetic textures and an out-of-plane ferroelectric polarization. The strong magnetoelectric coupling displayed by this twisted multiferroic allows for the manipulation of skyrmion lattice phases via an external electric field. These findings highlight the potential of twisted bilayer NiI_2_ for exploring unconventional magnetism and ultimately enabling spintronics devices that exploit electric-field control of topological spin textures.

## Supporting Information Available

Details of numerical calculation of the reported spin ground states, density functional theory calculations of interlayer exchange modulation, effects of anisotropic symmetric exchange and relaxation, and details on the effects of external out-of-plane electric fields (PDF)
