# Supplementary material for: Electric Field Control Of Moiré Skyrmion Phases in Twisted Multiferroic NiI2 Bilayers
Source: Nano Lett. 2024 Nov 22;24(49):15767–73. doi: 10.1021/acs.nanolett.4c04582 (PMC11638957; doi:10.1021/acs.nanolett.4c04582)
Supplement: Supplementary file 1 — nl4c04582_si_001.pdf [file nl4c04582_si_001.pdf]

Supplemental Information: Electric field control of moiré skyrmion phases in twisted multiferroic NiI_2 bilayers

Tiago V. C. Antão,[1] Jose L. Lado,[1] and Adolfo O. Fumega[1]

[1]*Department of Applied Physics, Aalto University, 02150 Espoo, Finland*

(Dated: November 8, 2024)

DETAILS OF THE SPIN GROUND STATE CALCULATION

In this section, we provide a short description of the method utilized for the calculation of the spin ground state. The spin configuration is determined by minimizing the total energy using the Broyden–Fletcher–Goldfarb–Shanno (BFGS) algorithm, implemented in the SciPy Python library [S1]. This gradient-based algorithm identifies the energy minimum by determining the descent direction and preconditioning the gradient with the curvature of the energy functional, i.e. by computing the gradient of the classical spin Hamiltonian. This computation is performed explicitly via differential programming with JAX [S2]. To ensure robustness, the parameters are initialized randomly, and the minimizations are repeated on the order of 200 times, with the minimum energy configuration being selected as the final one. We verify that different random initializations consistently lead to equivalent ground state configurations.

For the study of electric field control, in the case of adiabatic field evolution, the spin configuration is minimized randomly 200 times for the initial spin configuration, and subsequent minimizations at finite fields are performed iteratively. In each iteration, the electric field magnitude is increased by a small step, and the spin configurations are converged, taking as starting parameters the previous step's spin configurations. For non-adiabatic changes, the spin configuration is minimized from random initial conditions for both zero and large field intensities.

ROLE OF ADDITIONAL TERMS AND EFFECTS

In this section we analyze the role of additional terms or effects that can enter into the Hamiltonian for twisted NiI_2 bilayer and generally showcase that they provide little quantitative effect on the predicted phase diagram, pointing to the topologically protected nature of the computed phase diagram in the main text.

Symmetric anisotropic exchange

In the main text, a Hamiltonian is considered which includes a single-ion anisotropy term that results in an in-plane spiral configuration for the monolayer and is taken as the minimal term that allows to account for experimental observations [S3, S4]. Other anisotropic terms, such as a two-site anisotropic symmetric exchange will play a role in determining the chiral phase observed in the monolayer material by changing the magnitude of frustration as well as generating a spin spiral with a small out-of-plane component, which may be present in the monolayer ground-state spin spiral. For this reason, we focus on its effect on the predicted topological phases. Theoretically, this interaction can be expressed generically using a tensor representation as

$$\overline{\overline{J}}_{\text{ani}} = \begin{pmatrix} J_{xx} & J_{xy} & J_{xz} \\ J_{xy} & J_{yy} & J_{yz} \\ J_{xz} & J_{yz} & J_{zz} \end{pmatrix}, \tag{1}$$

with a corresponding term in the Hamiltonian given by

$$H_{\text{ani}} = \sum_{\langle i,j \rangle} \mathbf{S}_i \cdot \overline{\overline{J}}_{\text{ani}} \cdot \mathbf{S}_j \tag{2}$$

For a NiI_2 monolayer, a previous work [S5] reports $J_{xx}/J_1 \approx 0.14, J_{yy}/J_1 \approx -J_{yz}/J_1 \approx 0.20$ and $J_{zz}/J_1 \approx -0.04$, together with a value of $J_3/J_1 \approx 0.82$, which is much higher than $J_3/J_1 \approx 0.31$ extracted from with atomic-scale STM experiments performed on monolayer NiI_2[S4]. In contrast, our manuscript takes the value of J_3/J_1 consistent with experiments[S4]. Adding an anisotropic term of the magnitude suggested in Ref. [S5] while keeping the value of J_3 utilized in the main text would result in a ferromagnetic phase for the monolayer. This would therefore be inconsistent with the multiferroic nature of monolayer NiI_2 that has been found in more recent experiments[S4, S6]. For this reason, we scale the anisotropic interaction values to the third neighbor exchange interaction J_3 utilized in the main text, arriving at $J_{xx}/J_1 \approx 0.05, J_{yy}/J_1 \approx -J_{yz}/J_1 \approx 0.07$ and $J_{zz}/J_1 \approx -0.01$.

In Fig. S1 we present the ground state spin configuration for the skyrmion and skyrmionium lattice phases with and without the anisotropic symmetric exchange term. We find that although such terms result in some slight renormalization of the spin-spiral wavelength, and subsequently affect its competition with the moiré wavelength, these changes tend to be

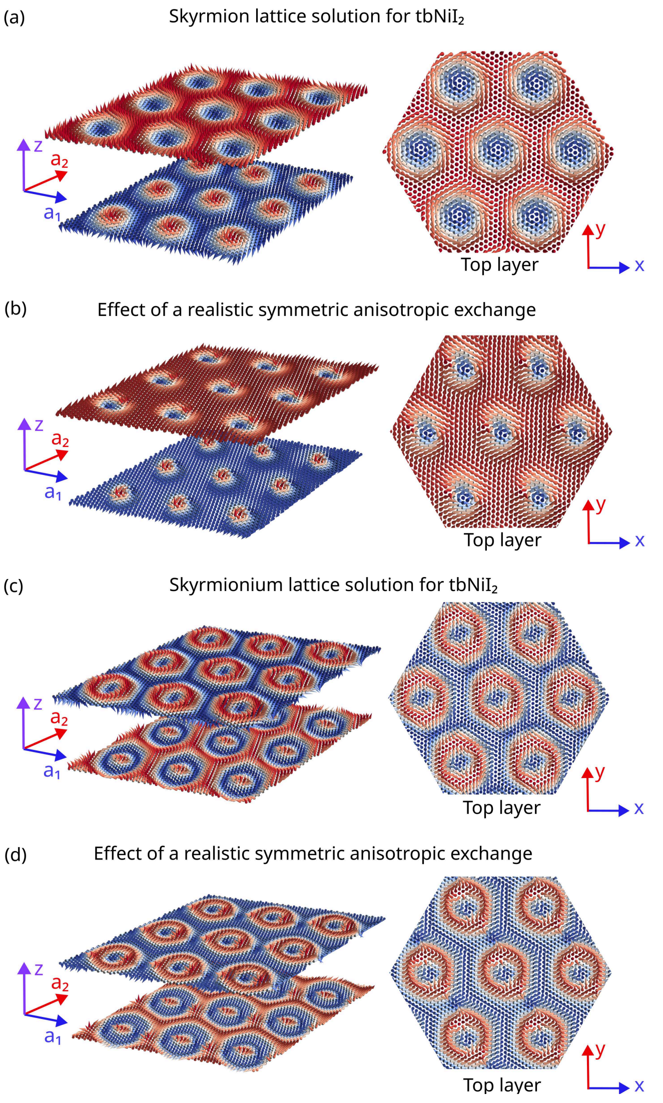

FIG. S1. (a) Skyrmion lattice solution obtained for a twist angle of $\theta \approx 3.9^\circ$ without symmetric anisotropic exchange. (b) Effects of the symmetric anisotropic exchange on the skyrmion lattice solution. Spins become preferentially aligned in a particular direction in space, while the skyrmion core becomes smaller. (c) Skyrmionium or $2\pi-$skyrmion lattice solution obtained for a twist angle of $\theta \approx 3.1^\circ$. (d) Ground state solution for the $2\pi-$skyrmion lattice in the presence of the anisotropic symmetric exchange. The spiral vector for the solution is also renormalized with more prominently aligned spins outside the skyrmionium core.

quantitatively small, and therefore they would result only in small deviations from the predicted phase diagram. Consistently with the expected phenomenology, for $k\pi$-skyrmion phases, these terms result in an overall tilting of spins outside the skyrmion's radius and shrinking of the skyrmion's core, breaking the perfect C_6 rotational symmetry. However, topological invariants and the $k\pi$ value is found to be preserved even in the presence of these terms.

Stacking modulation of interlayer exchange

In this section, we detail our *ab-initio* calculations as a function of stacking between the layers and introduce the predicted effects as a modulation in the interlayer exchange interactions of the moiré system.

We have performed ab initio Density Functional Theory (DFT) calculations with the all-electron full-potential linearized augmented-plane-wave method as implemented in the Elk code [S7]. We have used the PBE exchange-correlation functional and we have included an on-site interaction of $U = 3$ meV, consistent with previous first principles studies on NiI_2, where the [S8]. The results presented are converged with respect to all the parameters. In particular, a $9 \times 9 \times 1$ k-mesh, $R_{kmax} = 7$, and a vacuum spacing of 20 Åwere used to avoid interactions between neighboring cells in the z direction. Figure S2a shows the energy difference $\Delta E = E_{\mathrm{FM}} - E_{\mathrm{AFM}}$ between ferromagnetic and antiferromagnetic configurations for the two NiI_2 layers as a function of the stacking displacement vector d between the layers. The proportionality between this energy difference and the interlayer magnetic exchange interaction allows us to extract this parameter as a function of the local stacking. From the calculation shown in Fig. S2a, it is clear that the interlayer exchange does not change sign and remains antiferromagnetic for any stacking, only a small modulation is displayed. This result is in agreement with the other independent DFT calculations reported recently [S9]. We can now provide a mapping to the interlayer exchange interactions in the twisted bilayer system by changing the value of $J_\perp$ to a function of position

$$J_\perp = J_\perp^{(0)} V(\boldsymbol{r}), \tag{3}$$

where $V(\boldsymbol{r})$ corresponds to the relative value of energy difference or exchange interactions as estimated from DFT. The form of $V(\boldsymbol{r})$ is obtained numerically by mapping the local displacement of the moiré pattern to the relative energy difference obtained from the DFT results S2c. We can observe that the relative exchange modulation takes values up to 20% with respect to the mean value of the interlayer exchange.

Fig. S3 shows the effect of including the stacking-dependent modulation in the interlayer exchange $J_\perp$ as obtained from DFT to explore how this modulation influences the skyrmion phases. It can be seen in Fig. S3 that both the skymion lattice and the skyrmionium lattice phases undergo only small quantitative corrections, such as changing the radius of the skyrmion lattices. Topological features are preserved even for large modulations. This result justifies using a the functional of the interlayer exchange interaction presented in our manuscript. In other twisted van der

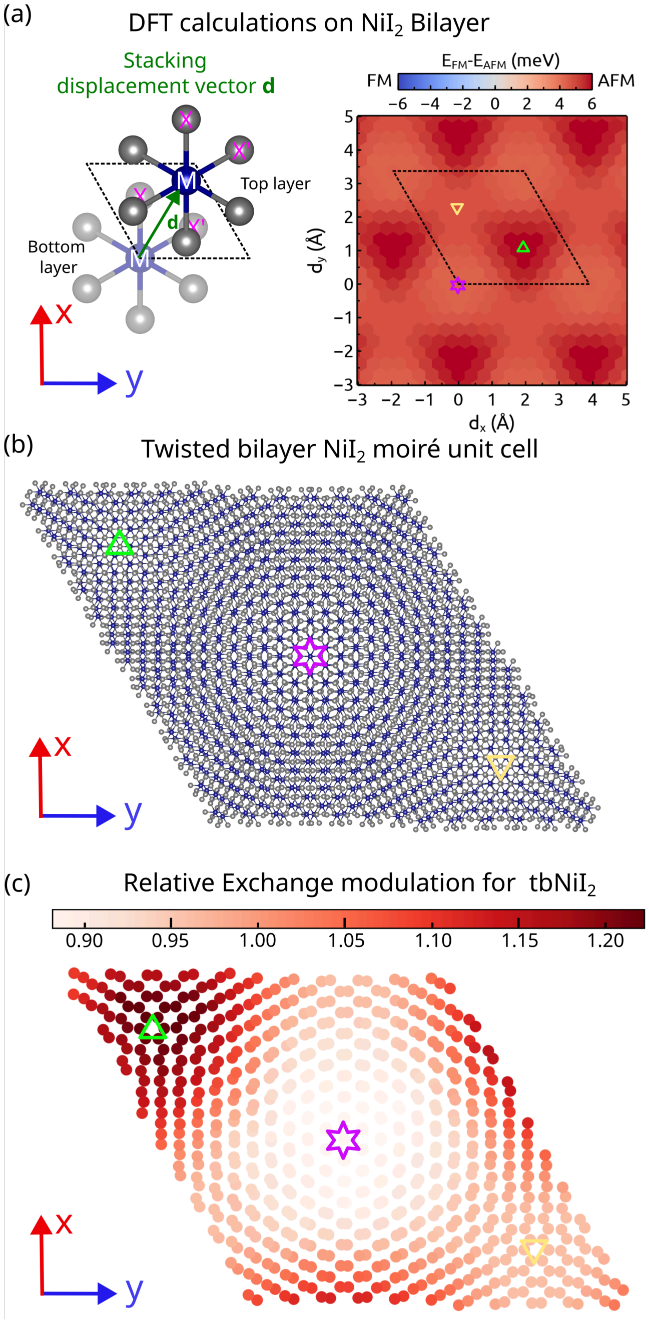

FIG. S2. (a) DFT calculations of the energy difference between ferromagnetic and antiferromagnetic interlayer alignment as a function of stacking. On the left, the displacement unit cell is represented, with the bottom layer placed at the origin. A displacement vector $\boldsymbol{d}$ (marked in green) is then applied to the top layer. The energy difference $\Delta E = E_{\rm FM} - E_{\rm AFM}$ is then computed as a function of the displacement between layers and showcased in the right panel. The displacement corresponding to an MM stacking with overlayed Nickel atoms is highlighted with a pink star, and MX and MX' stackings, corresponding to overlayed Nickel and Iodine atoms with green and yellow triangles, respectively. (b) Moiré pattern produced by the twisted bilayer NiI_2, with highlighted MM, MX and MX' local stacking regions. (c) Relative modulation of the exchange field in twisted NiI_2 as estimated from the energy difference obtained in the DFT calculations.

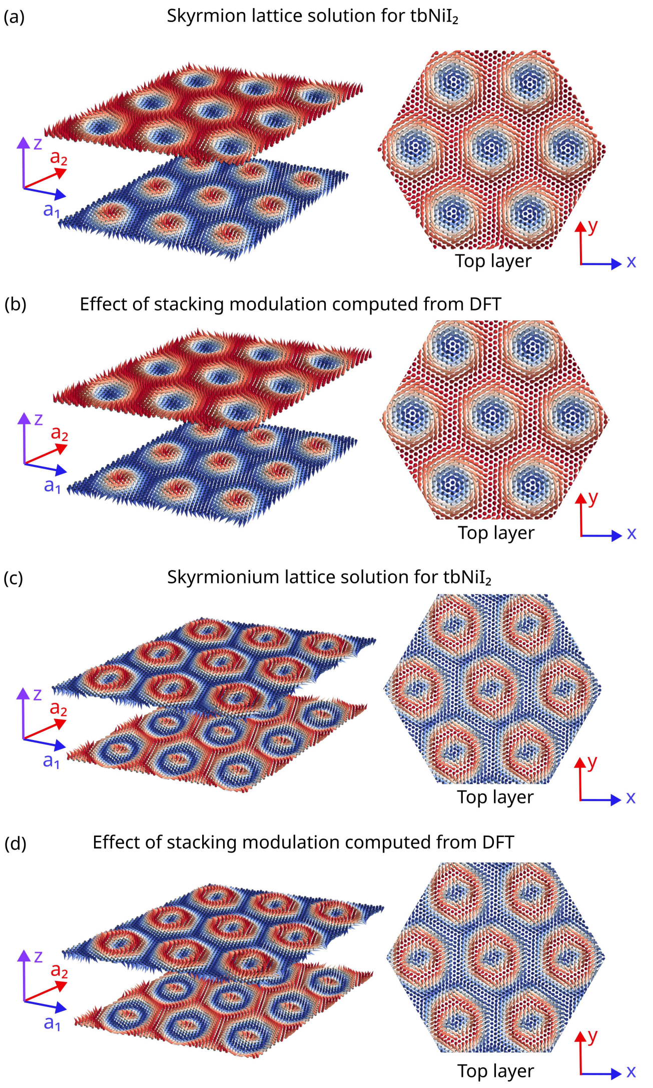

FIG. S3. (a) Skyrmion lattice solution obtained for a twist angle of $\theta \approx 3.9^\circ$ without stacking modulation (b) Effects of the stacking modulation on the interlayer exchange (c) Skyrmionium ($2\pi-$skyrmion) lattice solution, obtained for a twist angle of $\theta \approx 3.1^\circ$. (d) Effects of the stacking modulation on the interlayer exchange. No appeciable difference is verified for a relative modulation on the order of 25%. The directionality of the coupling for these interactions remains the same, and the modulation occurs in a way that does not significantly affect the symmetry of the interactions, with the C_2 symmetric regions where the local $\boldsymbol{q}-$vector is pinned (showcased in Fig. 1. and 2 of the main text) retaining relatively high exchange interactions.

Waals magnets such as $CrBr_3$, the sign of $J_\perp$ has a stacking dependence [S10–S12] that drives the different non-collinear orders. In stark contrast, NiI_2 features a coupling that does not change sign with the stacking. In addition, this finding further supports the notion that the emergent topological orders in twisted bilayer NiI_2 are not primarily induced by modulations in the interlayer

exchange, but rather by the interaction between the spin-spiral and moiré length scales.

Relaxation effects

In this section, in order to address the possible effects of relaxation, we proceed in an effective manner by mapping local displacements to a local modulation of the intralayer interactions. To take into account the effects of relaxation, we consider two different scenarios where the pattern of atomic displacements is consistent with a triple-q torsional pattern, expected from realistic relaxation models [S13]. In the first scenario, the MM stacking regions are contracted, while MX and MX' regions are expanded. The other complementary relaxation scenario corresponds to the expansion of the MM stacking regions and contraction of MX and MX'. In both cases, a displacement map is expected to obey

$$u(\boldsymbol{r}) = A\left(\cos(\boldsymbol{q}_1\cdot\boldsymbol{r}) + \cos(\boldsymbol{q}_2\cdot\boldsymbol{r}) + \cos(\boldsymbol{q}_3\cdot\boldsymbol{r})\right), \quad (4)$$

where A corresponds to the magnitude of displacements, and $\boldsymbol{q}_i$, with $i = 1,2,3$ correspond to the reciprocal moiré lattice vectors $\boldsymbol{q}_i \propto \boldsymbol{b}_i$. The effect of this relaxation on the exchange field can then be parametrized as

$$J_{ij} = J_{ij}^{(0)} e^{\pm u(\boldsymbol{r}_i)/\Gamma}, \quad (5)$$

where Γ is the decay factor on the order of unity, and a maximum displacement amplitude $\Delta u = 0.05a$ and $\Delta u = 0.1a$, such that the exchange field features a modulation on the order of 5% and 10% respectively [S13]. The choice of sign in the exponent of 5 is such that a $+(-)$sign corresponds to contracting(expanding) the MM region and expanding(contracting) the MX and MX' regions.

Our results point to the fact that such effects may work to slightly modulate the local $\boldsymbol{q}$-vector within the moiré unit cell, providing very small quantitative differences to the presented phase diagram, but do not introduce any qualitative changes to the predicted skyrmion phases. A further next step for future studies would be to take into account relaxation effects at the level of *ab initio* calculations, and we hope that our results motivate future work in this direction. It is finally worth noting that a relaxation consistent with the symmetry provided here which contracts the MX and MX' regions may work to further pin the skyrmionic texture to the lattice, since the exchange interactions within the C_2 symmetric stacking regions are also increased, promoting the mechanism for the pinning of skyrmionic textures described in the main text.

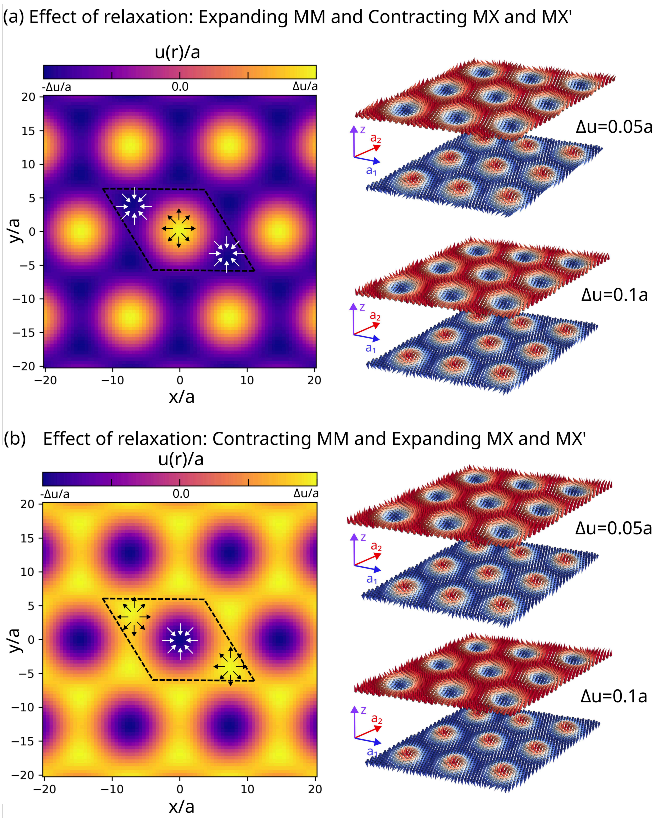

FIG. S4. Effect of relaxation on the spin configuration. The left panels show the effective relaxation parametrization $u(\boldsymbol{r})$ representing a maximum contraction and expansion by a factor of Δu which is chosen to match experimentally reasonable yet relatively large values of $\Delta u = 0.05a$ and $\Delta u = 0.1a$, on the order of $\Delta u \sim 20$ pm and $\Delta u \sim 40$ pm. The right panels showcase the effect of the modulation of $J_\perp$ by this relaxation on the spin configurations. (a) Relaxation patterns consist of expansion of the MM stacking region, leading to a local decrease in the value of $J_\perp$, and contraction of the MX and MX' regions, leading to a local increase in $J_\perp$. (b) Opposite relaxation pattern, with contraction of MM stacking region and expansion of MX and MX' regions. No significant differences are visible even in the presence of modulations of $J_\perp$ on the order of 20% as imposed by the large imposed relaxation modulations.

FIELD-INDUCED DZYALOSHINKII-MORIYA INTERACTION AND INTERPLAY OF LENGTH SCALES

In the main text we showcased that an electric field induces a Dzyaloshinskii-Moriya interaction (DMI) term via the magnetoelectric coupling Hamiltonian

$$H_{\mathrm{ME}} = \sum_{\langle ij\rangle} \boldsymbol{E}_\perp\cdot\boldsymbol{P}_{ij} = -\lambda E_\perp \sum_{\langle ij\rangle} (\hat{\boldsymbol{z}}\times\boldsymbol{r}_{ij})\cdot(\boldsymbol{S}_i\times\boldsymbol{S}_j)\,. \quad (6)$$

.

In this section we further explore the effect of the DMI and its competition with other length scales of the

system. This can be written explicitly in the form of a DMI using $\boldsymbol{D}_{ij} = \lambda E_\perp \hat{\boldsymbol{z}} \times \boldsymbol{r}_{ij}$ as

$$H_{\mathrm{ME}} = \sum_{\langle ij \rangle} \boldsymbol{D}_{ij} \cdot (\boldsymbol{S}_i \times \boldsymbol{S}_j) \, . \tag{7}$$

The DMI induces a new length-scale into the problem, determined by the magnitude of the vector $\boldsymbol{D}_{ij}$, which is directly proportional to the electric field intensity $E_\perp$. Here, we showcase additional calculations of the spin ground state in the twisted bilayer system for the case of a skyrmion lattice (i.e. $qL_m/2\pi = 1$) for a different moiré unit cell size, yielding a different relationship between q and L_m. This allows one to further explore the relationship between L_m and the internal length scales set by the magnetic interactions. Specifically, we can compare the evolution of the skyrmion lattice as a function of the electric field (or electric-field-induced DMI) for different moiré length scales.

Figure S5 shows the adiabatic evolution of the skyrmion lattice ($qL_m/2\pi = 1$) for the choice of parameters within this smaller moiré unit cell. The calculation presented in the main text corresponds to the particular case of bilayer NiI_2 ($q = 0.33/a$ and $L_m = 18.9a$) corresponds to the case of an average local $\boldsymbol{q}-$vector magnitude of $q = 0.45/a$ and a moiré length $L_m = 14.7a$.

In the main text, we can see that the adiabatic evolution in the larger L_m smoothly modifies the shape of the skyrmion from a perfectly C_6-symmetric skyrmion to a horseshoe shaped skyrmion, such that the winding $k\pi$ of the skyrmion is preserved. However, for the smaller moiré unit cell this horseshoe deformation does not occur, since the smaller L_m value is found to favour instead the formation of the three-fold symmetric stars. In this case, the C_6-symmetric skyrmion keeps its shape while resulting in an increase of its core's radius. At high enough electric field values, the originally C_6-symmetric skyrmion abruptly modifies its shape to a triangular star shape (S5). Importantly, this change in shape does not modify the winding $k\pi$ of the skyrmion lattice, which remains, up to deformations of the skyrmion core in the $k = 1$ phase. This analysis allows one to further rationalize the competition of electric-field-induced DMI, J_3/J_1 ratio (or q) and L_m. Generically, the role of the DMI is to provide an enhancement of non-collinearity, i.e. an increase in the magnitude of all local $\boldsymbol{q}$-vectors (Fig. S5). However, the hysteretic behavior of its evolution under adiabatic changes in the electric field signals the emergence of different topologically equivalent phases. For the skyrmion lattice of twisted bilayer NiI_2 discussed in the main text (Fig. S5), this leads to the emergence of horseshoe skyrmion phases with a smoother increase in the magnitude of all local $\boldsymbol{q}$-vectors as a function of the electric-field-induced DMI. This reflects a higher plasticity of the phases within the moiré unit cell owing to its larger size and higher number of novel deformed skyrmion configurations. In contrast, for the skyrmion lattice of the system with a larger q and smaller L_m (Fig. S5), the local $\boldsymbol{q}$-vectors evolve smoothly as a function of the electric-field-induced DMI until the transition to the triangular star shape occurs accompanied by a jump in the magnitude of all local $\boldsymbol{q}$-vectors (Fig. S5). This change in shape occurs at values of $q(E_\perp)L_m$ that allow for novel yet topologically equivalent configurations of the spin ground state, thus highlighting the interplay between the DMI enhanced $\boldsymbol{q}-$vector magnitude and L_m.

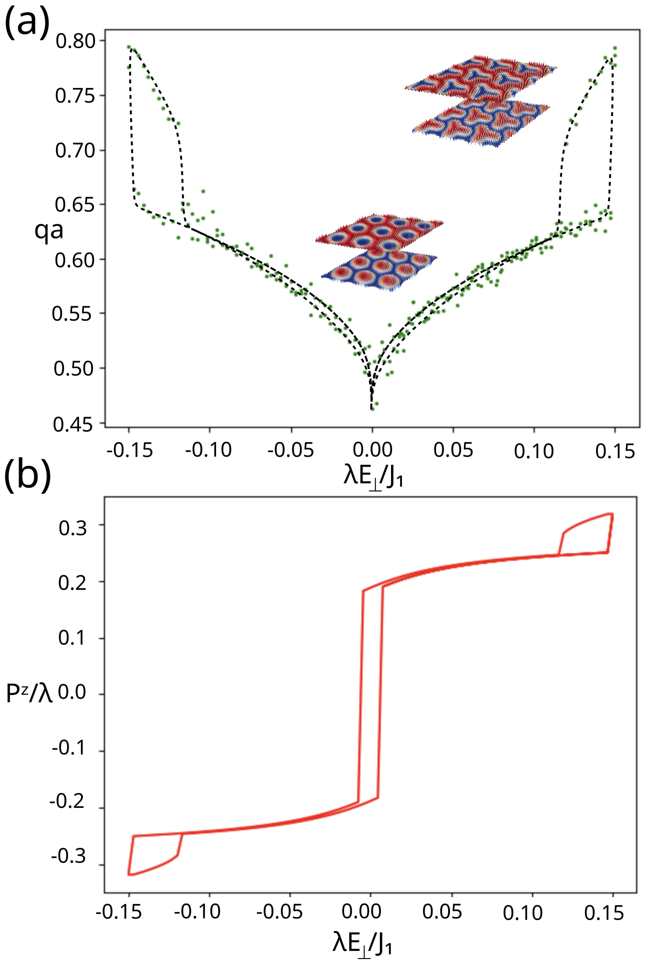

FIG. S5. (a) Average magnitude of local $\boldsymbol{q}-$vector as a function of adiabatic evolution of electric field in the cycle $\lambda E_\perp/J_1 = 0 \to 0.15 \to -0.15 \to 0$. Inset are the ground state skyrmion lattice ground states, together with a distorted three-fold symmetric skyrmion lattice which results in hysteretic behavior near a field of $\lambda E_\perp/J_1 \approx 0.125$. (b) Average polarization along z as a function of electric field in the same polarization cycle. The profile of the generated hysteresis loop is odd, but its absolute value is identical in shape to the curve observed for the average $\boldsymbol{q}-$vector. This points to the fact that the out-of-plane polarization can be used as a probe for the length scale associated with the magnetic textures, stemming from both DMI and J_3/J_1 ratio.

To summarize, the different evolution of skyrmion lattices with different q and L_m values as a function of the electric-field-induced DMI reflects the underlying competition of $\boldsymbol{q}$, determined by between the J_3/J_1 ratio and the DMI, and L_m. Furthermore, the magnitude of the local $\boldsymbol{q}-$vector is observed to be proportional to the absolute value of the emergent out-of-plane polarization, and hence we predict that measurements of the polarization can provide insights into the length scales of the system, with hysteretic behavior corresponding to signatures of competition between $\boldsymbol{q}$ and L_m.

REFERENCES

[S1] "Scipy python library," `https://scipy.org/`, (accessed 2024-17-01).

[S2] "Jax," `https://github.com/google/jax`, (accessed 2024-17-01).

[S3] Chengkun Song, Yunxu Ma, Chendong Jin, Jinshuai Wang, Haiyan Xia, Jianbo Wang, and Qingfang Liu, "Field-tuned spin excitation spectrum of k skyrmion," New Journal of Physics **21**, 083006 (2019).

[S4] Mohammad Amini, Adolfo O. Fumega, Héctor González-Herrero, Viliam Vaňo, Shawulienu Kezilebieke, Jose L. Lado, and Peter Liljeroth, "Atomic-scale visualization of multiferroicity in monolayer nii2," Advanced Materials **36** (2024), 10.1002/adma.202311342.

[S5] Danila Amoroso, Paolo Barone, and Silvia Picozzi, "Spontaneous skyrmionic lattice from anisotropic symmetric exchange in a ni-halide monolayer," Nature Communications **11** (2020), 10.1038/s41467-020-19535-w.

[S6] Qian Song, Connor A. Occhialini, Emre Ergeçen, Batyr Ilyas, Danila Amoroso, Paolo Barone, Jesse Kapeghian, Kenji Watanabe, Takashi Taniguchi, Antia S. Botana, Silvia Picozzi, Nuh Gedik, and Riccardo Comin, "Evidence for a single-layer van der waals multiferroic," Nature **602**, 601–605 (2022).

[S7] "The elk code," `https://elk.sourceforge.io/`, (accessed 2024-17-01).

[S8] Jesse Kapeghian, Danila Amoroso, Connor A. Occhialini, Luiz G. P. Martins, Qian Song, Jesse S. Smith, Joshua J. Sanchez, Jing Kong, Riccardo Comin, Paolo Barone, Bertrand Dupé, Matthieu J. Verstraete, and Antia S. Botana, "Effects of pressure on the electronic and magnetic properties of bulk nii_2," Phys. Rev. B **109**, 014403 (2024).

[S9] Daniel Bennett, Gabriel Martínez-Carracedo, Xu He, Jaime Ferrer, Philippe Ghosez, Riccardo Comin, and Efthimios Kaxiras, "Stacking-engineered ferroelectricity and multiferroic order in van der waals magnets," arXiv e-prints (2024), 10.48550/arXiv.2405.20069, (accessed 2024-10-02).

[S10] Adolfo O Fumega and Jose L Lado, "Moiré-driven multiferroic order in twisted crcl3, crbr3 and cri3 bilayers," 2D Materials **10**, 025026 (2023).

[S11] Muhammad Akram, Harrison LaBollita, Dibyendu Dey, Jesse Kapeghian, Onur Erten, and Antia S. Botana, "Moiré skyrmions and chiral magnetic phases in twisted CrX_3 (x = i, br, and cl) bilayers," Nano Letters **21**, 6633–6639 (2021).

[S12] Muhammad Akram, Jesse Kapeghian, Jyotirish Das, Roser Valentí, Antia S. Botana, and Onur Erten, "Theory of moiré magnetism in twisted bilayer α-rucl3," Nano Letters **24**, 890–896 (2024).

[S13] Suk Hyun Sung, Yin Min Goh, Hyobin Yoo, Rebecca Engelke, Hongchao Xie, Kuan Zhang, Zidong Li, Andrew Ye, Parag B. Deotare, Ellad B. Tadmor, Andrew J. Mannix, Jiwoong Park, Liyuan Zhao, Philip Kim, and Robert Hovden, "Torsional periodic lattice distortions and diffraction of twisted 2d materials," Nature Communications **13** (2022), 10.1038/s41467-022-35477-x.
